# Methods for Collection of Extracellular Vesicles and Their Content RNA as Liquid Biopsy for Lung Cancer Detection: Application of Differential Centrifugation and Annexin A5 Coated Beads

**DOI:** 10.3390/cimb44050162

**Published:** 2022-05-23

**Authors:** Mei-Chia Wang, Guan-Yu Gong, Chih-Liang Wang, How-Wen Ko, Rong-Xuan Weng, Pi-Yueh Chang, Chiuan-Chian Chiou

**Affiliations:** 1 Department of Laboratory Medicine, Chang Gung Memorial Hospital at Linkou, Taoyuan 333, Taiwan; ottermika@cgmh.org.tw (M.-C.W.); changpy@cgmh.org.tw (P.-Y.C.); 2 Department of Medical Biotechnology and Laboratory Science, College of Medicine, Chang Gung University, Taoyuan 333, Taiwan; e75967259@livemail.tw; 3 Graduate Institute of Biomedical Sciences, College of Medicine, Chang Gung University, Taoyuan 333, Taiwan; 4 Department of Thoracic Medicine, Chang-Gung Memorial Hospital at Linkou, Taoyuan 333, Taiwan; wang@cgmh.org.tw (C.-L.W.); howwenko@gmail.com (H.-W.K.); 5 Master and PhD Program in Biotechnolgy Industry, College of Medicine, Chang Gung University, Taoyuan 333, Taiwan; ken010194@gmail.com

**Keywords:** extracellular vesicles, annexin A5 beads, extracellular RNA, liquid biopsy, non-small-cell lung cancer

## Abstract

Extracellular vesicles (EVs) contain abundant extracellular RNA (exRNA), which can be a valuable source of liquid biopsy. However, as various RNA species exist in different types of EVs, lack of detailed characterization of these RNA species and efficient collection methods limits the clinical application of exRNA. In the present study, we measured two mRNAs, CK19 and PCTK1; one lncRNA, MALAT1; and two miRNAs, miR21 and miR155, in different EV fractions separated by differential centrifugation or captured by magnetic beads coated with annexin A5 (ANX beads). The results showed that in a cultured medium, the majority of mRNA and lncRNA exist in larger EVs, whereas miRNA exist in both large and small EVs from the differential centrifugation fractions. All these RNA species exist in ANX beads captured EVs. We then used ANX beads to capture EVs in plasma samples from non-small-cell lung cancer patients and age-matched healthy volunteers. We found that the ANX bead capturing could efficiently improve RNA detection from human plasma, compared with direct extraction of RNA from plasma. Using ANX-bead capturing and reverse transcription and quantitative PCR, we detected significantly higher levels of CK19 mRNA, MALAT1 lncRNA, and miR155 miRNA in the plasma of lung cancer patients. These facts suggested the collection methods strongly affect the results of exRNA measurement from EVs, and that ANX beads can be a useful tool for detecting exRNA from plasma samples in clinical application.

## 1. Introduction

Extracellular vesicles (EVs) are membranous vesicles derived from endosomal or plasma membrane and may play important roles in intercellular communication [1,2,3]. Recently, EVs have drawn intensive attention due to their possible application as a source of liquid biopsy for disease detection [4]. According to their origin and size, EVs can be classified into at least three types: exosomes (40–200 nm in diameter), microvesicles (50–1000 nm in diameter), and apoptotic bodies (50–5000 nm in diameter). Some researchers believe that cancer cells may also release a specific type of EV, namely oncosomes (50–5000 nm in diameter) [4]. These EVs have different formation pathways. For example, microvesicles are budded from membrane surface; exosomes are formed from multiendosomes; apoptotic bodies are blebbed from apoptotic cells. These different formation paths make them engulf different cell contents.

EVs contain various macro-molecules carrying information from the original cells [5,6,7]. Extracellular RNA (exRNA) is an important category of macromolecules in EVs. As exRNA is unstable in body fluids, it must be protected within lipid bilayer membrane or associated with proteins [8]. Various RNA species can be encapsulated in the EVs. For example, microRNA (miRNA) (76.2%), long noncoding RNA (lncRNA) (3.36%), and messenger RNA (mRNA) (1.36%) have been found in exosomes [9,10]. RNA cargo of EVs reflects the levels and types of cytoplasmic content, which provides clue of physiologic state of the cells releasing them [11]. Measurement of exRNA in EVs in serum or plasma have been explored as an approach of blood-based cancer detection [12,13,14,15,16,17]. However, a lack of reproducible and efficient approaches for collecting different EVs and exRNA limits its application [18].

Several methods have been proposed for EV collection and classification, usually dependent on size, membrane property or surface markers of EVs. These methods included centrifugation, affinity column, and microfluidic technology [19,20]. The most frequently used method for different EV isolation is differential centrifugation, which includes at least two steps of centrifugation: a low-speed centrifugation for pelleting larger EVs and a high-speed centrifugation for pelleting smaller EVs [21,22,23]. The second centrifugation is performed on an ultra-centrifuge and usually needs a high working volume [24]. The required speed and time are various in different laboratories [25]. Alternatively, some polymers can be added to facilitate precipitation of EVs, thus an ultra-centrifuge is undemanding [21]. Affinity column is another method to collect EVs, which applies a column specifically binding to membrane vesicles [26,27]. The common workflow of affinity purification is binding, washing, and elution. A centrifuge is also required in the procedures [26]. In general, these methods are either costly or time consuming. Most importantly, the requirement of centrifugation steps makes them difficult to fit in the laboratory automation system for clinical application.

In animal cells, the lipid phosphatidylserine (PS) is present in the inner leaflets of the plasma membrane [28]. When cells undergo apoptosis, PS translocates to the outer leaflets. In addition, PS is also found on the outer surface of the membrane of most EVs, especially apoptotic bodies and exosomes [29,30]. Annexin A5 is a calcium-dependent, PS binding protein. Thus, annexin A5 can be a useful agent for the recognition and collection of EVs. Shih et al. have developed an annexin A5-coated magnetic beads (ANX beads)-based procedure to capture EVs from fluidic samples [31]. The ANX beads had high affinity to the EVs, the spiked apoptotic bodies or endogenous EVs could be recovered from fluidic samples. They also showed that the captured EVs contained amplifiable RNA [31]. As this method does not need any sophisticated instrument or complicated operation, it can be easily adapted in an automation system, such as a magnetic particle processor. However, the EVs captured by the ANX beads need further characterization before the ANX beads can be used in clinical practice. In this study, we employed differential centrifugation and ANX beads methods to concentrate EVs from culture media and human plasma. The size and RNA content of the EVs were analyzed and compared. Then we applied the ANX bead method to collect exRNA and checked the diagnostic value of different RNA species in lung cancer.

## 2. Materials and Methods

### 2.1. Preparation of ANX Beads

The preparation of ANX beads has been described elsewhere [31]. Briefly, a human annexin A5 gene with a His-tag was cloned and expressed in *Escherichia coli*. An aliquot of bacterial lysate was mixed with MagneHis Ni (Promega, Madison, WI, USA) particles in a binding buffer containing 100 mM HEPES (pH 7.5), 10 mM imidazole, and 300 mM NaCl for 1 h at 4 °C to allow for an association between the annexin A5 protein and the magnetic beads through His-tag. The beads were washed with and stored in the binding buffer for subsequent use. 

### 2.2. Preparation of EVs from Cultured Cells

EVs may be secreted by living cells or shed from the bleb of apoptotic cells. We generated these two populations of EVs from cultured cells. H1299 human NSCLC cells were fed with RPMI1640 medium supplemented with 10% (*v/v*) fetal bovine serum, 100 U/mL penicillin/streptomycin, and 0.03% L-glutamate (Thermo Fisher Scientific, Waltham, MA, USA) and maintained at 37 °C and 5% CO_2_ in a humidified incubator. The cells were grown in T25 flasks until reaching a number around 1 × 10^6^ cells. To prepare EVs from living cells, the growth medium was replaced with fresh serum-free RPMI1640 medium for 24 h and the cell-free culture medium was collected. To prepare EVs from apoptotic cells, the medium was replaced with fresh RPMI1640 medium containing 5 µg/mL camptothecin (Sigma-Aldrich, St. Louis, MO, USA) and 3 µg/mL 5-fluorouracil (Sigma-Aldrich). After two days, the supernatant of the medium was collected. The quantities of EVs were expressed by their protein content, which was determined with a Bio-Rad Dc protein assay (Bio-Rad Laboratories, Hercules, CA, USA) after the lysis of the EVs with Tris buffered saline (pH 8.0) containing 1% NP-40 (Sigma-Aldrich).

### 2.3. Differential Centrifugation

Different sized EVs were separated with a two-step centrifugation protocol [32]: 500 μL culture mediums or plasma were centrifuged at 4 °C for 20 min at 16,000× *g* to separate pellet (DCF1) and supernatant. The supernatant was transferred to a new tube and centrifugated for a second time at 120,000× *g* for 70 min. The second pellet (DCF2) and supernatant (DCF3) were collected. Before further analysis, the DCF1 and DCF2 were resuspended in phosphate-buffered saline (PBS).

### 2.4. Capture of EVs by ANX Beads 

A 500-μL aliquot of the medium with EVs was mixed with 500 μL Ca-HEPES buffer (containing 10 mM HEPES, pH 7.5; 1 mM MgCl_2_; 5 mM KCl; 2% BSA; 15 mM CaCl_2_; and 150 mM NaCl) and ANX beads. The mixture was gently agitated on a rotator for 15 min at 4 °C and then incubated without agitating for 30 min at room temperature to capture EVs. The EV–bead complexes were then pulled down by a magnetic stand and the supernatant was removed. The beads were washed twice and resuspended with 500 µL Ca-HEPES buffer [31].

### 2.5. Nanoparticle Tracking Analysis (NTA)

The pellets collected from differential centrifugation (DCF1 and DCF2) were resuspend with 500 µL PBS. ANX beads concentrated EVs were eluted by 500 µL EDTA elution buffer. Nanoparticle tracking analysis was performed with a NanoSight NS300 (Malvern Panalytical, Malvern, UK) equipped with a 488 nm laser to measure the number and size distribution of the EVs. Video capture was performed at camera level 15, slider shutter 1206, slider gain 366 for 60 s intervals. A total of 1498 captures were taken for each sample 3 times. Samples were analyzed with appropriate threshold setting. Video capture and analysis was carried out using NanoSight NTA software 3.4 (Malvern Panalytical).

### 2.6. RNA Processing and Analysis

Total nucleic acid was extracted using LabTurbo Viral Nucleic Acid Extraction kits (Labturbo Biotech, Taipei, Taiwan) from a 300 µL culture medium or plasma sample. The samples were mixed with a 300 µL lysis buffer and 30 µL proteinase K, then incubated at 56 °C for 10 min and processed on an automatic LabTurbo 24/48 Compact System (Labturbo Biotech). This method generated 60 µL of purified nucleic acid solution. Total RNA concentration was measured using Qubit RNA HS Assay Kits on a Qubit 4.0 Fluorometer (Thermo Fisher Scientific). Specific RNAs were measured by reverse transcription-quantitative polymerase chain reaction (RT-qPCR), using commercial kits from Thermo Fisher Scientific. For mRNA and lncRNA, random hexamers were used as reverse transcription (RT) primers. For miRNA, gene-specific loop primers were used as RT primers. RNA was converted into cDNA using SuperScript III First Strand Synthesis Supermix (Thermo Fisher Scientific). The cDNA was amplified with target-specific primers and probes using TaqMan Gene Expression Assays (Thermo Fisher Scientific), performed on a QuantStudio 12K Flex Real-Time PCR System (Thermo Fisher Scientific). The TaqMan Gene Expression Assays included CK19 (Hs00761767_s1), PCTK1 (Hs00178837_m1), MALAT-1 (Hs00273907_s1), GAPDH (Hs002786624_g1), and β2M (Hs00984230_m1). The TaqMan MiRNA Assay included miR21 (467534_mat) and miR155 (000397). After RT-qPCR, the threshold cycle (Ct) was obtained for each reaction and was used to estimate gene expression level. For the experiments with multiple measurements, a batch of standard RNA from cultured cells was subjected to each assay and its Ct value was used to normalize the measurement. The RNA level of each gene was thus expressed as delta Ct, in which delta Ct = Ct_standard_ − Ct_gene_.

### 2.7. Patients and Clinical Samples

Twenty healthy volunteers and 22 NSCLC patients were recruited at Chang Gung Memorial Hospital, Taiwan. This study was approved by the Institute Review Board of Chang Gung Memorial Hospital (approval number 201801557B0). Peripheral blood from the healthy volunteers and the patients was collected in tubes containing EDTA as anticoagulants. Blood samples were centrifuged at 530× *g* for 10 min to separate plasma. An aliquot of 500-μL plasma was mixed with 500 μL Ca-HEPES buffer and ANX beads. The mixture was gently agitated on a rotator for 15 min at 4 °C, and then incubated without agitating for 30 min at room temperature to capture EVs. The EV–bead complexes were then pulled down by a magnetic stand and the supernatant was removed. The beads were washed twice with a 1 mL Ca-HEPES buffer and resuspended in a 300 µL LabTurbo lysis buffer and 30 µL proteinase K, then incubated in 56 °C for 10 min. RNA in the sample was extracted on the automatic LabTurbo 24/48 Compact System. Total RNA concentration was measured using a Qubit Fluorometer and specific RNAs were measured by RT-qPCR, as described in Section 2.6.

### 2.8. Statistical Analysis

Student’s *t*-test was used for statistical analysis. Results were considered significant at *p* < 0.05. The receiver operating characteristic curve was made using SigmaPlot 10 Software.

## 3. Results

### 3.1. Collection and Characterization of EVs Separated by Differential Centrifugation 

**Figure 1:**
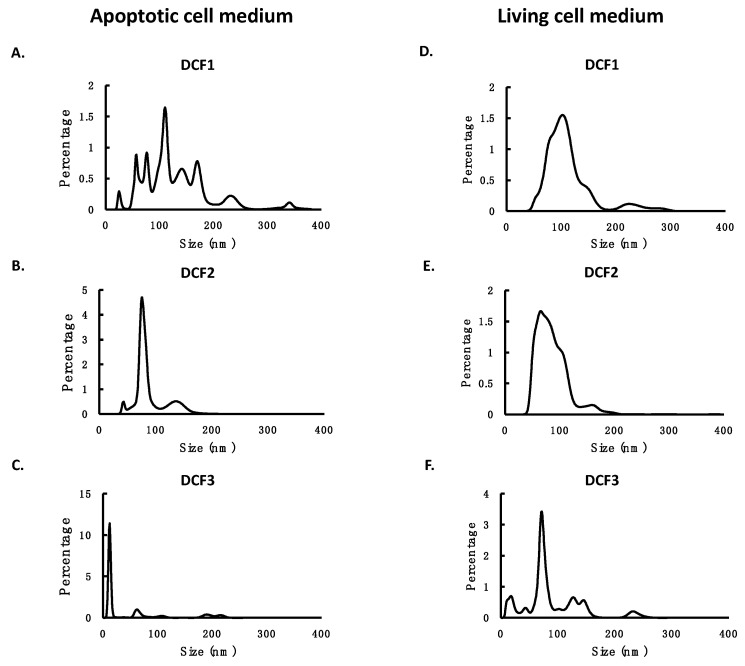
The particle size profiles of different differential centrifugation fractions of EVs from the medium with apoptotic cells (**A**–**C**) and that with living cells (**D**–**F**). DCF1, DCF2, and DCF3 were three fractions of the media derived from two-step centrifugation, as described in Materials and Methods.

EVs may be secreted by living cells or shed from bleb of apoptotic cells. We first generated these two populations of EVs by collecting the culture medium with living cells or the medium with apoptotic cells. Then we applied differential centrifugation to fractionate the media using a two-step protocol: a first centrifugation at 16,000× *g* for 20 min, followed by a second ultra-speed centrifugation at 120,000× *g* for 70 min. This protocol divided EVs into three fractions. The pellet of the first centrifugation was designated as DCF1, which was expected to consist mostly of larger particles; the pellet of the second centrifugation was designated as DCF2, which was expected to contain smaller particles; and the supernatant of the second centrifugation was designated as DCF3, which was expected to consist of soluble molecules, very small EVs, or nonmembrane particles like protein aggregates or protein-RNA complexes. Then we applied nanoparticle tracking analysis (NTA) to measure the number and size of EVs in these fractions. The results showed that in the medium with living cells, the most abundant particles were small particles that exist in DCF2, whereas in the medium with apoptotic cells, the most abundant particles were large particles that exist in DCF1 (Table 1). The NTA profiles confirmed this observation and showed the tendency of particle size: DCF1 > DCF2 > DCF3 (Figure 1). The majority of EVs in DCF1 were particles larger than 100 nm in diameter, whereas those in DCF2 and DCF3 were particles smaller than 100 nm in diameter (Table 1 and Figure 1). 

### 3.2. Different RNA Species Distributed in Different Differential Centrifugation Fractions

**Figure 2:**
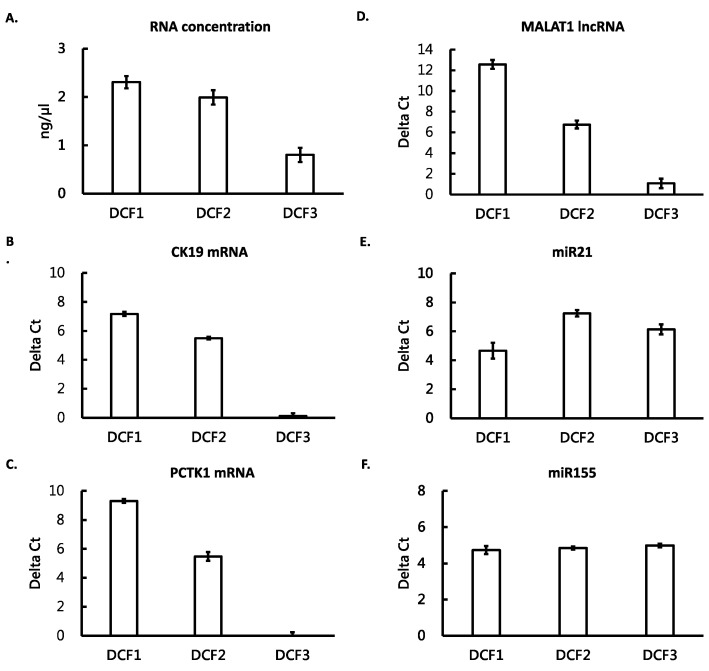
RNA contents in different DC fractions of EVs from medium with living cells. DCF1, DCF2, and DCF3 were three fractions of the media derived from two-step centrifugation, as described in Materials and Methods. RNA was purified from the fractions and its concentration was determined by a Qubit fluorometer (**A**). The levels of different RNA species were measured by RT-qPCR. These RNA species included mRNAs of CK19 and PCTK1 (**B**,**C**, respectively), lncRNA of MALAT1 (**D**), and miRNAs of miR21 and miR155 (**E**,**F**, respectively). All the measurements were performed in triplicate and the bars are shown as mean ± standard error.

One of the purposes of the present study was to understand the distribution of different RNA species in different differential centrifugation fractions; we selected five RNA biomarkers from three RNA species as targets for this purpose. These RNA biomarkers have been reported to be associated with lung cancer, including CK19 and PCTK1 mRNAs [31,33,34], MALAT1 lncRNA [35,36], and miR21 and miR155 miRNAs [37,38,39,40]. The different differential centrifugation fractions from the medium with living cells was subjected to RNA extraction and RNA quantification. The levels of different RNA species were measured by reverse transcription-quantitative polymerase chain reaction (RT-qPCR). The results showed that DCF1 and DCF2 contained higher concentration of total RNA than DCF3 (Figure 2A). The majority of the two mRNAs and the lncRNA distributed mainly in DCF1, lesser in DCF2, and almost none in DCF3 (Figure 2B–D). In contrast, the two miRNAs distributed more evenly, i.e., they were in similar levels in DCF1, DCF2, and DCF3 (Figure 2E,F). These results indicated that the mRNAs and the lncRNA were allocated mainly in larger particles, whereas the mi-RNAs exist in all differential centrifugation fractions.

### 3.3. ANX Beads Captured Different Sized EVs

**Figure 3:**
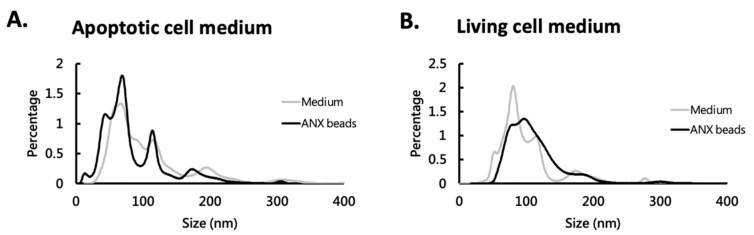
Comparison of particle size profile of the EVs in medium and that captured by ANX beads. The EVs in cultured medium with apoptotic cells (**A**) and living cells (**B**) were either analyzed directly by nanoparticle tracking analysis (NTA) or captured with ANX beads, eluted, and analyzed by NTA.

We have previously shown that ANX beads could capture EVs from conditioned medium and human plasma [31]. In the present study, we further characterized the captured EVs from media with living cells and media with apoptotic cells. The media were first mixed with ANX beads to let EVs be captured, and then the captured EVs were eluted and analyzed by NTA. The results showed that the ANX beads could capture large (>100 nm) and small (<100 nm) particles (Table 1). From either the medium with apoptotic cells or that with living cells, the size profile of the captured EVs were similar to that in the media (Figure 3). The NTA data showed that the size of ANX bead-captured EVs ranged from 19.5 to 571.5 nm in apoptotic cell medium and from 45.5 to 653.5 nm in living cell medium. This fact suggested that these different sized EVs can associate with the ANX beads. 

### 3.4. ANX Beads Recovered RNA from Medium and Human Plasma 

**Figure 4:**
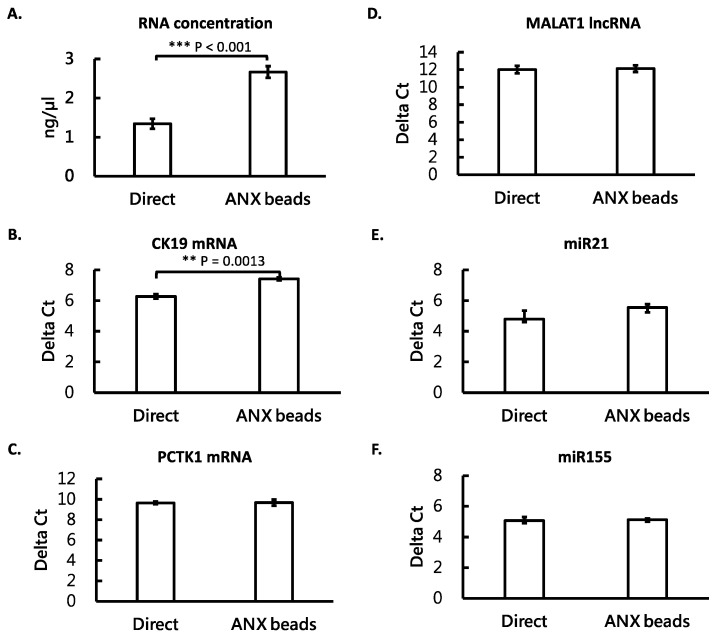
ANX beads could recover RNA from cultured medium. The media with living cells were either subjected to RNA extraction directly or first mixed with ANX beads to capture EVs and then subjected to RNA extraction. Total RNA concentration was determined by a fluorometer (**A**). The levels of different RNA species were measured by RT-qPCR. These RNA species included mRNAs-CK19 and PCTK1 (**B**,**C**, respectively), lncRNA-MALAT1 (**D**), and miRNAs-miR21 and miR155 (**E**,**F**, respectively). All the measurements were performed in triplicate and the bars are shown as mean ± standard error. Student’s *t* test was used to compare the groups. ** *p* < 0.01; *** *p* < 0.001.

**Figure 5:**
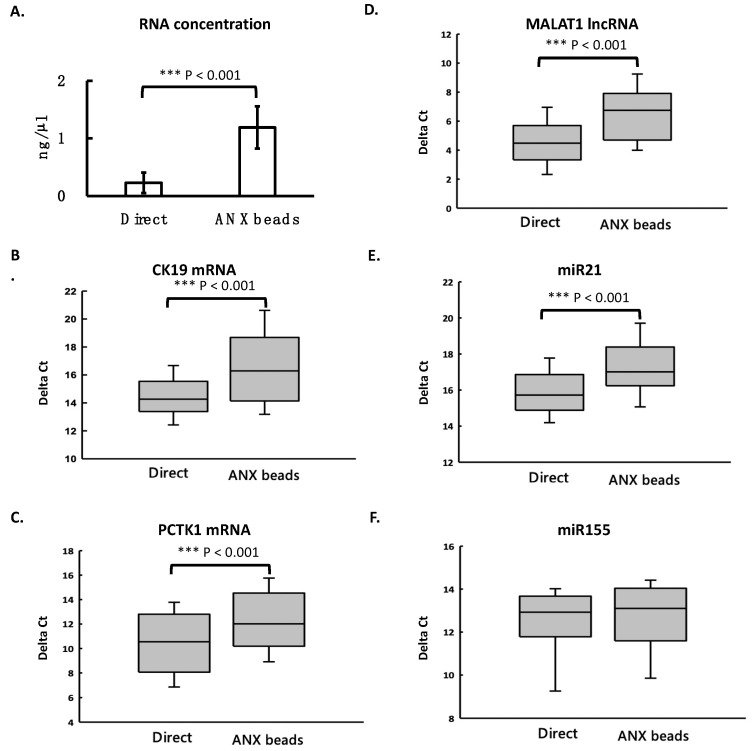
ANX beads increased exRNA recovery from plasma samples. The plasma samples (n = 50) were either subjected to RNA extraction directly or first mixed with ANX beads to capture EVs and then subjected to RNA extraction. Total RNA concentration was determined by a fluorometer (**A**). The levels of different RNA species were measured by RT-qPCR. These RNA species included mRNAs-CK19 and PCTK1 (**B**,**C**, respectively), lncRNA-MALAT1 (**D**), and miRNAs-miR21 and miR155 (**E**,**F**, respectively). Student’s *t*-test was used for statistical analysis. Results were considered significant at *p* < 0.05. *** *p* < 0.001.

We then compared the recovery of RNA extracted from the ANX bead-captured EVs and that extracted directly from the cultured medium with living cells. The results showed that after ANX bead capturing, the measured total RNA concentration was higher than that from direct purification (Figure 4A). However, the levels of mRNA, lncRNA, and miRNA were similar when using both methods (Figure 4B–F). Only CK19 mRNA was slightly higher in the ANX bead method (Figure 4B). Then the comparison was applied in human plasma samples. RNA from plasma was either extracted from the ANX bead-captured EVs or extracted directly from plasma. The results showed that the measured total RNA concentration was significantly higher than that from direct purification (Figure 5A). All the levels of different RNA species were also higher when using the ANX bead method except miR155 (Figure 5B–F). These results indicated that the ANX beads could efficiently recover exRNA from cultured medium and plasma samples. The reason why the ANX bead method seemed to have higher efficiency for recovering RNA will be discussed below.

### 3.5. Applying ANX Bead-Captured EV for Diagnosis of Lung Cancer 

**Figure 6:**
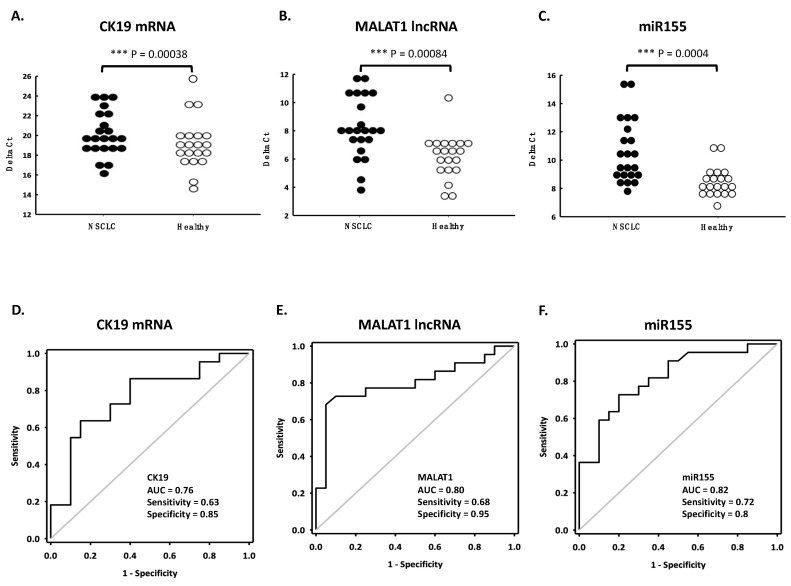
The diagnostic performance of circulating CK19 (**A**,**D**), MALAT1 (**B**,**E**) and miR155 (**C**,**F**) RNA for NSCLC using ANX beads. Plasma samples from NSCLC patients (n = 22) and healthy controls (n = 20) were subjected to RNA purification with ANX beads and RNA quantification with RT-qPCR. The diagram shows the distribution of the RNA levels of different groups (**A**–**C**) and their receiver operating characteristic curves (**D**–**F**). *** *p* < 0.001.

We then investigated whether the circulating exRNA could be used as cancer markers. Plasma samples from 22 non-small-cell lung cancer (NSCLC) patients and 20 age-matched healthy controls were collected for the study. RNA from the samples were extracted by ANX beads or directly from plasma. Five RNAs (CK19 mRNA, PCTK1 mRNA, MALAT1 lncRNA, miR155 miRNA, and miR21 miRNA) were determined by RT-qPCR. When using the RNA from ANX bead-captured EV, three RNAs had significantly higher levels in NSCLC patients then in healthy controls and showed good diagnostic value in the receiver operating characteristic (ROC) curve. They were CK19 mRNA (Figure 6A,D), MALAT1 lncRNA (Figure 6B,E), and miR155 miRNA (Figure 6C,F). The levels of PCTK1 mRNA and miR21 miRNA showed no difference between the two groups (Supplementary Figure S1). When using RNA directly extracted from plasma, the levels of four RNAs showed no differences between the two groups. Only miR155 had a better diagnostic value (Supplementary Figure S2). These facts suggested that the three circulating RNAs, CK19, MALAT1, and miR155 could be promising diagnostic markers for NSCLC when using ANX beads for EV capturing and RNA purification.

## 4. Discussion

In this study, we used differential centrifugation to separate EV fractions from cultured medium and measured three RNA species in these EVs. The differential centrifugation divided the EVs into three fractions, DCF1, DCF2, and DCF3. We found that in the three fractions, the majority of mRNA and lncRNA existed in DCF1 and DCF2, which contained mostly large and medium EVs, but not in DCF3, which contained very small particles or soluble molecules. However, miRNA could be detected in all three fractions. We also found that the ANX beads, which captured EVs with PS in the outer membrane, was able collect large and small EVs and detected the three RNA species in them. By using the ANX beads, we were able measure differential expression of CK19 mRNA, MALAT1 lncRNA, and miR155 miRNA in the plasma of NSCLC patients.

According to the size distribution of the three fractions (Table 1 and Figure 1), DCF1 contained most particles with sizes larger than 100 nm. Cell debris, apoptotic bodies, or microvesicles fall into this range. DCF2 contained particles with sizes around 100 nm. Microvesicles and exosomes are of this size. DCF3 contained particles with sizes much smaller than 100 nm. ExRNA in this fraction may be located in very tiny vesicles or associated with protein or lipid. Although we did not further classify specific types of EVs, the result that the majority of mRNA and lncRNA existed in DCF1 and DCF2 indicates the two RNA species existed mainly in large EVs but not in tiny EVs or protein-RNA or lipid-RNA complexes. This fact also suggests that procedures using intensive centrifugation or filtration may cause the loss of most mRNA and lncRNA and may not be suitable for the detection of these two RNA species.

In contrast to mRNA and lncRNA, miRNA existed in all the fractions, including DCF3. DCF3 contained soluble molecules and tiny particles that could not be pelleted by the ultra-speed centrifugation. These tiny particles may include very small EVs or aggregation of macromolecules. Some reports showed that miRNA can form protein-RNA complexes or lipid-RNA complexes in cultured medium or body fluids [8,41], which is consistent with our findings. The fact that miRNA can be detected in large EVs, small EVs, and molecular aggregations indicates that miRNA is a relatively abundant RNA species and exists in different conformations, which makes miRNA a good source of exRNA as a liquid biopsy. However, diseases may only change distribution of miRNA in certain types of EVs and choosing particular types of EVs may be important for the diagnostic purpose of miRNA.

In Figure 4 and Figure 5, the measured total RNA concentration was higher when using the ANX bead method than that of direct purification. This might be because the steps of EV capturing and washing in the ANX bead method efficiently removed some impurities which could not have been removed by the direct purification method. The impurities interfered with the measurement of RNA by the Qubit fluorescent method, resulting in a lower reading and underestimated concentration. The impurities seemed to not be an issue in the cultured medium, as the RT-qPCR detected similar levels of different RNA species extracted by both the ANX bead method and the direct purification method. However, in plasma samples, the existence of impurities was a more severe issue as the measured total RNA concentration and the levels of different RNA species were much higher when using the ANX bead method than when using direct purification method. These facts suggest that using ANX beads to capture EVs before RNA purification is a better procedure to detect circulating exRNA in plasma samples.

To our knowledge, we are the first to apply ANX beads in EV collection and exRNA detection in the blood of lung cancer patients. In addition to the removal of possible impurities, ANX beads provide many other advantageous features: Firstly, they can capture different types of EVs with PS expressed on the outer membrane. Secondly, ANX beads can concentrate EVs from large amounts of liquid biopsy. Thirdly, the procedure using magnetic beads can be easily incorporated into an all-in-one device for automatic EV capturing, RNA extraction, and quantitative PCR. In the future, exRNA biomarkers found in the present study have the potential to be combined with a biosensor to develop an inexpensive, point-of-care testing method, as is seen in the example of survivin mRNA [42,43]. We suggest that the ANX beads can be a powerful tool for circulating exRNA purification and disease detection.

## 5. Conclusions

Circulating exRNA has potential diagnostic value. However, different RNA species exist in different EVs and choosing suitable collection methods can be crucial for detecting certain types of RNA. The ANX bead method is an efficient tool for collecting EVs and their RNA contents from plasma samples and may be applied in the diagnosis of NSCLC.

## Figures and Tables

**Table 1:** The concentration and size distribution of particles fractionated by differential centrifugation or captured by ANX beads.

	No. of Particles (×10^8^/mL)	Small Particles (<100 nm) (%)	Large Particles (>100 nm) (%)
Living-cell medium			
DCF1	2.46	34.1	65.9
DCF2	5.46	66.2	33.8
DCF3	0.21	64.5	35.5
ANX-beads	4.96	23.1	76.9
Apoptotic-cell medium			
DCF1	2.11	21.5	78.5
DCF2	0.123	54.4	45.6
DCF3	0.0257	77.7	22.3
ANX beads	2.64	43.0	57.0

## Data Availability

The datasets used and/or analyzed during the current study are available from the corresponding author on reasonable request.

## Supplementary Materials

The following are available online at https://www.mdpi.com/article/10.3390/cimb44050162/s1, Supplementary Figure S1: When using ANX beads for RNA purification from plasma samples, the ROC curves of PCTK1 mRNA (A) and miR21 miRNA (B) showed no promising diagnostic value in 22 NSCLC patients and 20 healthy controls. Supplementary Figure S2: When using RNA directly purified from plasma samples, the ROC curves of all five RNA markers showed no promising diagnostic value in 22 NSCLC patients and 20 healthy controls.

## References

[1] Kim C.W., Lee H.M., Lee T.H., Kang C., Kleinman H.K., Gho Y.S. (2002). Extracellular membrane vesicles from tumor cells promote angiogenesis via sphingomyelin. Cancer Res..

[2] Yoon Y.J., Kim O.Y., Gho Y.S. (2014). Extracellular vesicles as emerging intercellular communicasomes. BMB Rep..

[3] Raposo G., Stoorvogel W. (2013). Extracellular vesicles: Exosomes, microvesicles, and friends. J. Cell Biol..

[4] Kosaka N., Kogure A., Yamamoto T., Urabe F., Usuba W., Prieto-Vila M., Ochiya T. (2019). Exploiting the message from cancer: The diagnostic value of extracellular vesicles for clinical applications. Exp. Mol. Med..

[5] Choi D.S., Kim D.K., Kim Y.K., Gho Y.S. (2013). Proteomics, transcriptomics and lipidomics of exosomes and ectosomes. Proteomics.

[6] Mayr M., Grainger D., Mayr U., Leroyer A.S., Leseche G., Sidibe A., Herbin O., Yin X., Gomes A., Madhu B. (2009). Proteomics, metabolomics, and immunomics on microparticles derived from human atherosclerotic plaques. Circ. Cardiovasc. Genet..

[7] Luz I., Cooks T. (2019). Extracellular vesicles: What secrets do they hold inside?. Cell Death Dis..

[8] Murillo O.D., Thistlethwaite W., Rozowsky J., Subramanian S.L., Lucero R., Shah N., Jackson A.R., Srinivasan S., Chung A., Laurent C.D. (2019). exRNA Atlas Analysis Reveals Distinct Extracellular RNA Cargo Types and Their Carriers Present across Human Biofluids. Cell.

[9] Huang X., Yuan T., Tschannen M., Sun Z., Jacob H., Du M., Liang M., Dittmar R.L., Liu Y., Liang M. (2013). Characterization of human plasma-derived exosomal RNAs by deep sequencing. BMC Genom..

[10] Cocucci E., Meldolesi J. (2015). Ectosomes and exosomes: Shedding the confusion between extracellular vesicles. Trends Cell Biol..

[11] Abels E.R., Breakefield X.O. (2016). Introduction to Extracellular Vesicles: Biogenesis, RNA Cargo Selection, Content, Release, and Uptake. Cell Mol. Neurobiol..

[12] Silva J., Garcia V., Rodriguez M., Compte M., Cisneros E., Veguillas P., Garcia J.M., Dominguez G., Campos-Martin Y., Cuevas J. (2012). Analysis of exosome release and its prognostic value in human colorectal cancer. Genes Chromosomes Cancer.

[13] Skog J., Würdinger T., van Rijn S., Meijer D.H., Gainche L., Sena-Esteves M., Curry W.T., Carter B.S., Krichevsky A.M., Breakefield X.O. (2008). Glioblastoma microvesicles transport RNA and proteins that promote tumour growth and provide diagnostic biomarkers. Nat. Cell Biol..

[14] Tanaka Y., Kamohara H., Kinoshita K., Kurashige J., Ishimoto T., Iwatsuki M., Watanabe M., Baba H. (2013). Clinical impact of serum exosomal microRNA-21 as a clinical biomarker in human esophageal squamous cell carcinoma. Cancer.

[15] Kosaka N., Iguchi H., Ochiya T. (2010). Circulating microRNA in body fluid: A new potential biomarker for cancer diagnosis and prognosis. Cancer Sci..

[16] Nilsson J., Skog J., Nordstrand A., Baranov V., Mincheva-Nilsson L., Breakefield X.O., Widmark A. (2009). Prostate cancer-derived urine exosomes: A novel approach to biomarkers for prostate cancer. Br. J. Cancer.

[17] Taylor D.D., Gercel-Taylor C. (2008). MicroRNA signatures of tumor-derived exosomes as diagnostic biomarkers of ovarian cancer. Gynecol. Oncol..

[18] Li K., Rodosthenous R.S., Kashanchi F., Gingeras T., Gould S.J., Kuo L.S., Kurre P., Lee H., Leonard J.N., Liu H. (2018). Advances, challenges, and opportunities in extracellular RNA biology: Insights from the NIH exRNA Strategic Workshop. JCI Insight.

[19] Kırbaş O.K., Bozkurt B.T., Asutay A.B., Mat B., Ozdemir B., Öztürkoğlu D., Ölmez H., İşlek Z., Şahin F., Taşlı P.N. (2019). Optimized Isolation of Extracellular Vesicles From Various Organic Sources Using Aqueous Two-Phase System. Sci. Rep..

[20] He M., Zeng Y. (2016). Microfluidic Exosome Analysis toward Liquid Biopsy for Cancer. J. Lab. Autom..

[21] Serrano-Pertierra E., Oliveira-Rodríguez M., Rivas M., Oliva P., Villafani J., Navarro A., Blanco-López M.C., Cernuda-Morollón E. (2019). Characterization of Plasma-Derived Extracellular Vesicles Isolated by Different Methods: A Comparison Study. Bioengineering.

[22] Lobb R.J., Becker M., Wen S.W., Wong C.S., Wiegmans A.P., Leimgruber A., Möller A. (2015). Optimized exosome isolation protocol for cell culture supernatant and human plasma. J. Extracell. Vesicles.

[23] Kalra H., Drummen G.P., Mathivanan S. (2016). Focus on Extracellular Vesicles: Introducing the Next Small Big Thing. Int. J. Mol. Sci..

[24] Konoshenko M.Y., Lekchnov E.A., Vlassov A.V., Laktionov P.P. (2018). Isolation of Extracellular Vesicles: General Methodologies and Latest Trends. Biomed. Res. Int..

[25] Yu L.L., Zhu J., Liu J.X., Jiang F., Ni W.K., Qu L.S., Ni R.Z., Lu C.H., Xiao M.B. (2018). A Comparison of Traditional and Novel Methods for the Separation of Exosomes from Human Samples. Biomed. Res. Int..

[26] Stranska R., Gysbrechts L., Wouters J., Vermeersch P., Bloch K., Dierickx D., Andrei G., Snoeck R. (2018). Comparison of membrane affinity-based method with size-exclusion chromatography for isolation of exosome-like vesicles from human plasma. J. Transl. Med..

[27] Enderle D., Spiel A., Coticchia C.M., Berghoff E., Mueller R., Schlumpberger M., Sprenger-Haussels M., Shaffer J.M., Lader E., Skog J. (2015). Characterization of RNA from Exosomes and Other Extracellular Vesicles Isolated by a Novel Spin Column-Based Method. PLoS ONE.

[28] Kay J.G., Fairn G.D. (2019). Distribution, dynamics and functional roles of phosphatidylserine within the cell. Cell Commun. Signal..

[29] Lima L.G., Chammas R., Monteiro R.Q., Moreira M.E., Barcinski M.A. (2009). Tumor-derived microvesicles modulate the establishment of metastatic melanoma in a phosphatidylserine-dependent manner. Cancer Lett..

[30] Subra C., Laulagnier K., Perret B., Record M. (2007). Exosome lipidomics unravels lipid sorting at the level of multivesicular bodies. Biochimie.

[31] Shih C.L., Chong K.Y., Hsu S.C., Chien H.J., Ma C.T., Chang J.W., Yu C.J., Chiou C.C. (2016). Development of a magnetic bead-based method for the collection of circulating extracellular vesicles. N Biotechnol..

[32] Bray F., Ferlay J., Soerjomataram I., Siegel R.L., Torre L.A., Jemal A. (2018). Global cancer statistics 2018: GLOBOCAN estimates of incidence and mortality worldwide for 36 cancers in 185 countries. CA Cancer J. Clin..

[33] Chen T.F., Jiang G.L., Fu X.L., Wang L.J., Qian H., Wu K.L., Zhao S. (2007). CK19 mRNA expression measured by reverse-transcription polymerase chain reaction (RT-PCR) in the peripheral blood of patients with non-small cell lung cancer treated by chemo-radiation: An independent prognostic factor. Lung Cancer.

[34] Yu X.F., Yang H.J., Lei L., Wang C., Huang J. (2016). CK19 mRNA in blood can predict non-sentinel lymph node metastasis in breast cancer. Oncotarget.

[35] Zhang R., Xia Y., Wang Z., Zheng J., Chen Y., Li X., Wang Y., Ming H. (2017). Serum long non coding RNA MALAT-1 protected by exosomes is up-regulated and promotes cell proliferation and migration in non-small cell lung cancer. Biochem. Biophys. Res. Commun..

[36] Ren S., Wang F., Shen J., Sun Y., Xu W., Lu J., Wei M., Xu C., Wu C., Zhang Z. (2013). Long non-coding RNA metastasis associated in lung adenocarcinoma transcript 1 derived miniRNA as a novel plasma-based biomarker for diagnosing prostate cancer. Eur. J. Cancer.

[37] Dejima H., Iinuma H., Kanaoka R., Matsutani N., Kawamura M. (2017). Exosomal microRNA in plasma as a non-invasive biomarker for the recurrence of non-small cell lung cancer. Oncol. Lett..

[38] Liu Q., Yu Z., Yuan S., Xie W., Li C., Hu Z., Xiang Y., Wu N., Wu L., Bai L. (2017). Circulating exosomal microRNAs as prognostic biomarkers for non-small-cell lung cancer. Oncotarget.

[39] Wei J., Gao W., Zhu C.J., Liu Y.Q., Mei Z., Cheng T., Shu Y.Q. (2011). Identification of plasma microRNA-21 as a biomarker for early detection and chemosensitivity of non-small cell lung cancer. Chin. J. Cancer.

[40] Roth C., Kasimir-Bauer S., Pantel K., Schwarzenbach H. (2011). Screening for circulating nucleic acids and caspase activity in the peripheral blood as potential diagnostic tools in lung cancer. Mol. Oncol..

[41] Dimitrova-Paternoga L., Jagtap P.K.A., Chen P.C., Hennig J. (2020). Integrative Structural Biology of Protein-RNA Complexes. Structure.

[42] Stobiecka M., Ratajczak K., Jakiela S. (2019). Toward early cancer detection: Focus on biosensing systems and biosensors for an anti-apoptotic protein survivin and survivin mRNA. Biosens. Bioelectron..

[43] Ratajczak K., Krazinski B.E., Kowalczyk A.E., Dworakowska B., Jakiela S., Stobiecka M. (2018). Hairpin-Hairpin Molecular Beacon Interactions for Detection of Survivin mRNA in Malignant SW480 Cells. ACS Appl. Mater. Interfaces.

