# Supplementary material for: Methods for Collection of Extracellular Vesicles and Their Content RNA as Liquid Biopsy for Lung Cancer Detection: Application of Differential Centrifugation and Annexin A5 Coated Beads
Source: Curr Issues Mol Biol. 2022 May 23;44(5):2374–86. doi: 10.3390/cimb44050162 (PMC9164077; doi:10.3390/cimb44050162)
Supplement: Supplementary file 1 [file cimb-44-00162-s001.zip › cimb-1719549-supplementary.pdf]

Supplementary Materials

Methods for Collection of Extracellular Vesicles and Their Content RNA as Liquid Biopsy for Lung Cancer Detection: Application of Differential Centrifugation and Annexin A5 Coated Beads

Mei-Chia Wang [1,2,3], Guan-Yu Gong [2], Chih-Liang Wang [4], How-Wen Ko [4], Rong-Xuan Weng [5], Pi-Yueh Chang [1,2] and Chiuan-Chian Chiou [2,3,4,5,*]

[1] Department of Laboratory Medicine, Chang Gung Memorial Hospital at Linkou, Taoyuan 333, Taiwan; ottermika@cgmh.org.tw (M.-C.W.); changpy@cgmh.org.tw (P.-Y.C.)
[2] Department of Medical Biotechnology and Laboratory Science, College of Medicine, Chang Gung University, Taoyuan 333, Taiwan; e75967259@livemail.tw
[3] Graduate Institute of Biomedical Sciences, College of Medicine, Chang Gung University, Taoyuan 333, Taiwan
[4] Department of Thoracic Medicine, Chang-Gung Memorial Hospital at Linkou, Taoyuan 333, Taiwan; wang@cgmh.org.tw (C.-L.W.); howwenko@gmail.com (H.-W.K.)
[5] Master and PhD Program in Biotechnolgy Industry, College of Medicine, Chang Gung University, Taoyuan 333, Taiwan; ken010194@gmail.com
* Correspondence: ccchiou@mail.cgu.edu.tw; Tel.: +886-3211-8800 (ext. 5204)

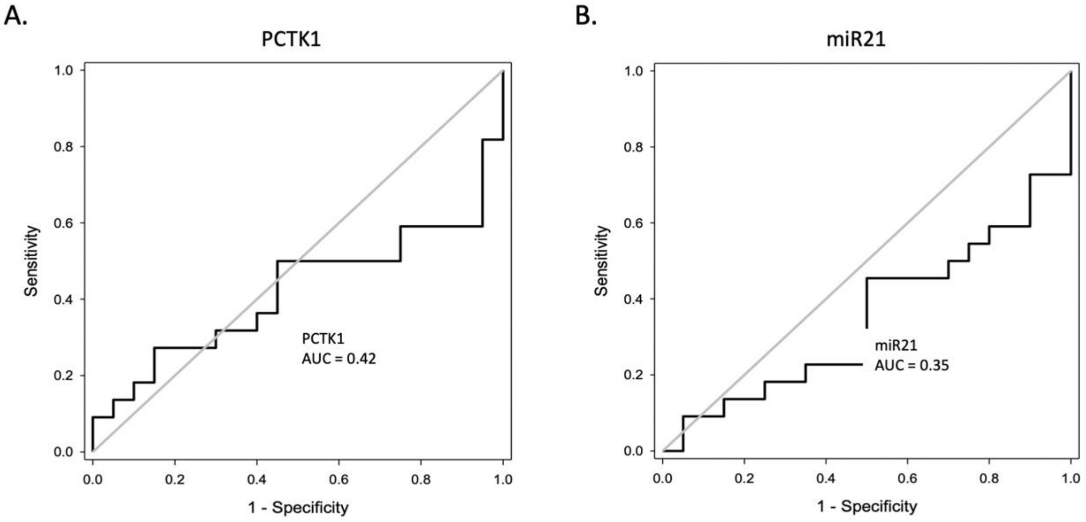

Figure S1. When using ANX beads for RNA purification from plasma samples, the ROC curves of PCTK1 mRNA (**A**) and miR21 microRNA (**B**) showed no promising diagnostic value in 22 NSCLC patients and 20 healthy controls.

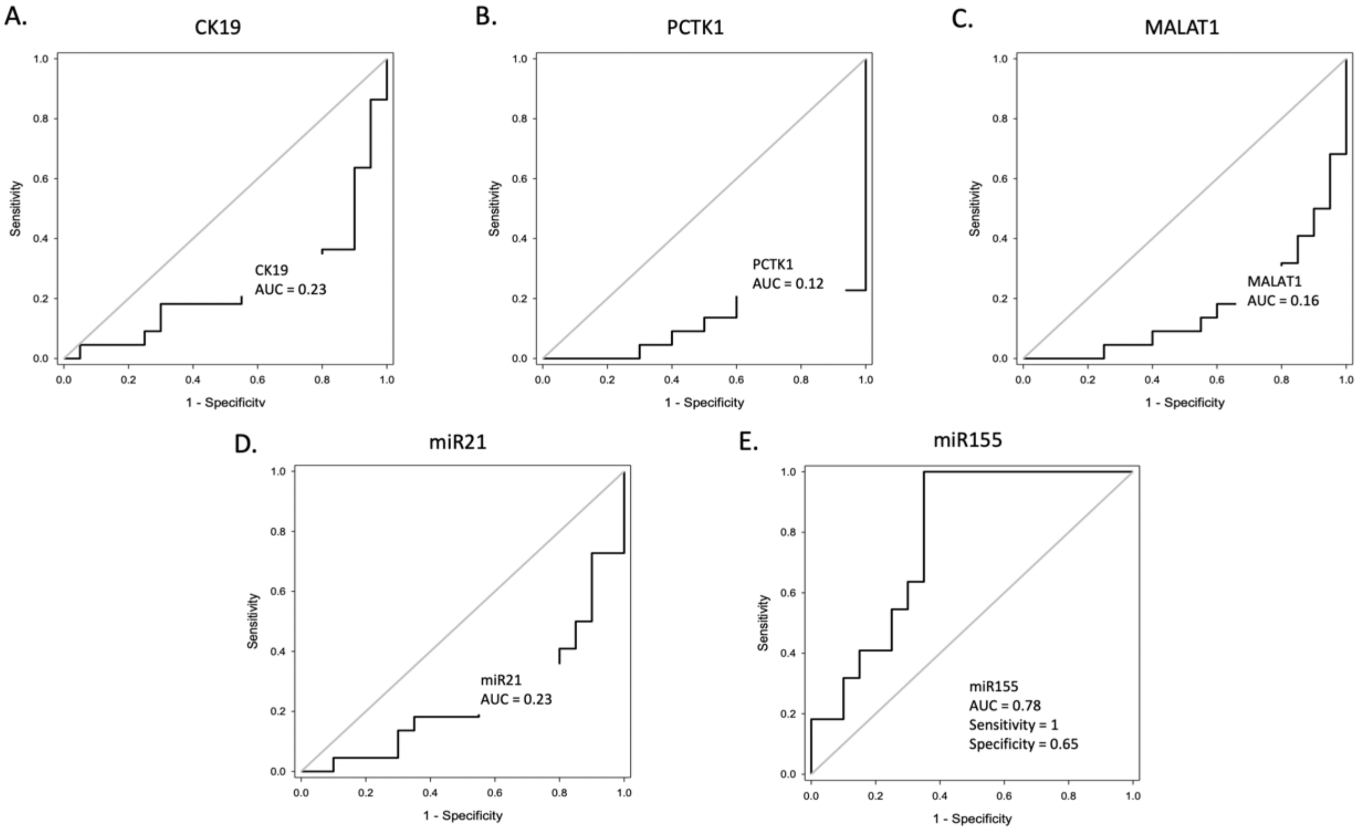

Figure S2. When using RNA directly purified from plasma samples, the ROC curves of all five RNA markers showed no promising diagnostic value in 22 NSCLC patients and 20 healthy controls.
